# Neurologic Complications of Influenza and Potential Protective Vaccine Effects

**DOI:** 10.1111/irv.70071

**Published:** 2025-03-06

**Authors:** Ann R. Falsey

**Affiliations:** ^1^ School of Medicine and Dentistry University of Rochester Rochester New York USA

**Keywords:** Alzheimer's disease, dementia, influenza, influenza vaccine, stroke

## Abstract

Influenza is a common respiratory infection affecting persons of all ages and results in significant morbidity and mortality. Respiratory complications are well known, but important nonpulmonary complications are less well recognized. Neurologic complications following influenza infection may accompany the acute illness or may be chronic in nature. The acute complications such as seizures, encephalitis, myelitis and Guillain Barre Syndrome are well documented but fortunately are uncommon. However, stroke and dementia are leading causes of death and disability worldwide, and there is increasing evidence linking these devasting illnesses with influenza. In addition, influenza vaccine has been associated with protective effects against stroke and dementia risk.

## 1 Introduction

Influenza is one of the most common afflictions of mankind resulting approximately 1 billion cases of seasonal influenza each year leading to 3–5 million hospitalizations and 290,000–650,000 deaths worldwide [1]. Similarly, neurologic disorders are among the most frequent causes of death and disability in world. In 2019, stroke and Alzheimer's disease (ad) and other dementias were listed as the second and seventh leading causes of death worldwide according to the World Health Organization [2]. The acute complications of seasonal and pandemic influenza have been well described in children and adults [3, 4, 5]. Stroke is now recognized as a potential complication of influenza and can cause acute disability but may also be linked to long‐term effects due to microvascular disease [6, 7, 8]. Finally, the long‐term effects of influenza infection may include subsequent development of Parkinson's disease, ad, or other forms of dementia [9]. Establishing a link between infectious agents and dementia is an area of active research and debate. This review will focus primarily on the possible chronic complications of influenza as well as evidence that influenza vaccination may be protective against these potentially devasting diseases.

## 2 Acute Neurologic Complications of Influenza

Although uncommon, acute neurologic complications of seasonal and pandemic influenza have been described and include delirium, psychosis with hallucinations, delusions, seizures, meningitis, encephalitis, myelitis, Reye's syndrome, acute disseminated encephalomyelitis and Guillain‐Barre syndrome [4, 5, 10, 11]. Fortunately, with the exception of delirium, which is generally self‐limited, these types of events are rare so that the burden of acute neurologic disease as a complication of influenza pales in comparison to the chronic complications such as stroke and dementia. The COVID‐19 pandemic has brought the neurologic complications of viral respiratory diseases to the forefront of medical attention. Notably, in a recent study using TriNetX, a global electronic medical record database, neurologic complications of influenza and SARS‐CoV‐2 were assessed up to 12 months following hospitalization [12]. Postinfection neurologic diagnoses occurred in 2.8% of COVID patients and 4.9% of influenza patients with a HR of 0.62 (95% CI 0.58–0.66) for COVID‐19 compared to influenza. The proportion of influenza patients recording encounters for migraine were 3.2%, epilepsy (2.1%), neuropathy (3.6%), movement disorders (2.5%), stroke (2.4%), and dementia (2.3%). These data highlight that patients hospitalized with influenza are at risk for subsequent neurologic events.

## 3 Parkinson's Disease

Historically, the first connection of influenza and chronic neurologic disease followed the H1N1 Spanish influenza pandemic [13]. Encephalitis lethargica was the term given to a type of postencephalitic Parkinson's (PEP) observed after the pandemic characterized by extreme sleepiness and in some cases coma and commonly associated with ocular muscle paresis and nystagmus [14]. It is estimated that approximately 2–3 million people developed PEP following the pandemic with this disorder gradually disappearing by the 1930s [15]. Neuropathologic findings revealed diffuse brain atrophy and neuronal loss in the substantia nigra and basal ganglia with neuronal and glial accumulation of tau proteins. Viral antigens in the hypothalamus and midbrain of patients with PEP have been inconsistently described [11, 15]. Notably, some individuals with long‐lasting PEP responded to levodopa treatment decades later, and this phenomenon was the subject of the 1990 movie “Awakenings.” No consensus was ever achieved as to the role of the 1918 influenza pandemic as the cause of encephalitis lethargica, but circumstantial evidence remains [16].

Apart from the 1918 influenza pandemic, Parkinson's disease remains a serious neurogenerative disease affecting more than 6 million people worldwide characterized by movement difficulty and cognitive decline [17]. Pathology involves cell death in the basal ganglia and decreased dopamine production. Recent epidemiologic studies have linked influenza infection with a subsequent increased risk of developing Parkinson's disease. A Danish case–control study from 1977 to 2016 analyzed 10,271 Parkinson's disease patients and 51,355 controls without Parkinson's disease [18]. A significant association of having an influenza diagnosis over 10 years prior and development of Parkinson's disease was noted with an odds ratio (OR) of 1.73 (95% CI 1.11–2.71). Using data from Finnish biobank and replication in a UK database, investigators examined the association of a variety of viral exposures with neurodegenerative disease risk [9]. Influenza with pneumonia was significantly associated with Parkinson's disease with HR for Parkinson's disease at 1–5 years post exposure of 2.24 and 1.72 at 10 years or greater similar to the Danish study.

Other literature using different size populations, study designs, and lag time between exposure and Parkinson's diagnosis offer conflicting results regarding risk of Parkinson's disease and prior influenza infection [19, 20, 21]. Lastly, data on the effect of influenza vaccination subsequent risk of Parkinson's disease are presently limited. Zhao et al. used a regression model adjusted for baseline and time varying covariates and found no protective effect of influenza vaccination and risk of Parkinson's disease [22].

Given the challenges of epidemiologic studies, animal models have shown promise to elucidate the role of influenza in chronic brain disease. Jang et al. found that H5N1 inoculated intranasally was capable of infecting CNS and induced neurodegenerative changes [23]. Notably, infection induced transient loss of the dopaminergic phenotype in the substantia nigra pars complex and basal ganglia with increased activation of microglial cells. Increased microglial activation may lead to over expression of α‐synuclein, a protein linked genetically and neuropathologically to Parkinson's disease [23].

Although there was a temporal relationship of the 1918 influenza pandemic and PEP, the role of seasonal influenza and subsequent risk of Parkinson's disease remains an unanswered question.

## 4 Influenza Infection and Risk of Stroke

The association of cardiovascular events (CVEs) and influenza epidemics has been recognized for over 90 years and was first reported in 1932 in *Public Health Reports* by Selwyn Collins, a statistician, who noted that there were excess noninfluenza and pneumonia deaths during periods of high influenza activity [24]. Influenza‐associated CVE initially was attributed to illness‐induced stress in patients with preexisting atherosclerotic disease; however, recent research has highlighted possible molecular mechanisms such as inflammatory cytokine release, prothrombotic states, and endothelial dysfunction [25]. Ecologic studies relating influenza activity and CVEs provided the first clues of this association [26, 27, 28, 29]. A recent example of this type of study is a time‐series analysis of English hospital admissions demonstrating a significant association of myocardial infarction and stroke with laboratory confirmed viral respiratory infections from 2004 to 2015 [30]. Importantly, in the adjusted model, all respiratory viruses including influenza were associated with ischemic stroke in persons over age 75 years.

Because ecologic studies have been criticized due to possible unmeasured bias, a new methodology called the self‐controlled case series (SCCS) has been a significant step forward in defining the influenza–CVE connection. In the SCCS design, cases act as their own controls, and thus, inherently control for inter‐individual variability [31, 32, 33, 34, 35]. In databases containing persons with both influenza infection and a first CVE (stroke or myocardial infarction), a baseline period and a period of risk after exposure are defined. Relative risk (RR) during baseline periods can then be compared to the period of risk. The first report using the SCCS methodology was in 2004 by Smeeth et al. who evaluated persons with first myocardial infarction or stroke and influenza vaccine or acute respiratory infections during the period of surveillance [7]. No increased risk of CVE was found after vaccination but the incident rate ratio (IRR) for stroke was greatest during the 1st 3 days after ARI at 3.19 (95% CI 2.81 to 3.63) and gradually decreasing to baseline over 90 days. Using similar methodology, Boehme et al. found that the OR of ischemic stroke was greatest Days 1–15 post influenza‐like illness (ILI) and diminished over time with no relationship evident by 60 days post ILI [31]. Interestingly, there was a significant interaction of age and ILI with the OR of stroke increasing with each 10‐year decrease in age such that the greatest OR (9.28) was in the 18‐ to 45‐year‐old group. Scottish, Danish, and US studies using SCCS analysis have all shown increased risk of stroke following influenza infection [6, 34, 35]. Increased rates of stroke after infection were generally highest with IRR of 4.0–10.3 in the first 1–7 days following infection but remained elevated to out to 28 days. The increased risk of stroke includes both ischemic and hemorrhagic strokes and may also be related to the severity of the influenza illnesses [6, 33].

## 5 Effect of Influenza Vaccination and Risk of Stroke

Epidemiological studies have suggested a significant inverse relationship between influenza vaccination and risk of fatal and nonfatal CVE [36, 37]. Evidence regarding prevention of stroke by influenza vaccine is largely based on observational case–control studies, which suggest a protective effect but are inconsistent and criticized for potential unmeasured bias [38, 39, 40, 41, 42, 43, 44]. Systematic reviews and meta‐analyses as well as SCCS studies are now offering more compelling support for the benefits of influenza vaccination as a measure to reduce risk of stroke [37, 44, 45, 46, 47, 48, 49].

Although observational, several recent case–control studies specifically addressing the effects of influenza vaccination and stroke have used propensity score matching (PSM) as well as logistic regression models to adjust for confounding clinical covariates [38, 41]. Canadian investigators used a database from 2009 through 2018 to evaluate the effect of recent influenza vaccine (< 182 days) and stroke in persons over age 18 years [38]. The study involved over 4 million persons of whom 73% received at least one influenza vaccination and demonstrated that the risk for all types of strokes was significantly reduced with HR of 0.78 (95% CI 0.76–0.79) with recent influenza vaccination. Rodriguez‐Martin et al. used a nested case–control study using a Spanish primary care database from 2001 to 2015 of people 44 to 99 years of age and matched 14,322 stroke patients (67% nonembolic and 33% embolic strokes) 5:1 to nonstroke control patients [41]. After adjustment for confounders, vaccine was associated with a moderate protective effect (OR 0.88 [95% CI 0.84–0.92]) appearing early at Days 15 to 30 and declining thereafter. Reduced risk of stroke with vaccine was observed in all subgroups and during all three epidemics. Notably, the effect was similar during preepidemic, epidemic, and postepidemic periods. Several additional studies have evaluated influenza and risk of stroke in subpopulations of interest such as atrial fibrillation and younger patients and have also demonstrated protective effects of vaccination [39, 42].

The SCCS study design has also been used to evaluate the protective effects of influenza vaccine. The first report using SCCS to evaluate influenza vaccination and risk of stroke was by Asghar et al. who computed incidence rate ratio (IRR) of stroke during fixed time periods after vaccination compared to baseline [48]. The incidence of stroke was significantly reduced in the first 59 days post vaccination with the strongest reduction of 55% during Days 1–3 post vaccination dropping to 17% reduction at 29 to 59 days. Early season vaccination had a stronger effect than late season vaccination.

Using a national database in England from 2008 to 2019 and a SCCS design, Davidson et al. evaluated 193,900 patients with CVE and influenza vaccination [40]. Stroke was reduced with IR of 0.70 at 15–28 days (95% CI 0.67–0.74) gradually decreasing to 0.87 (95% CI 0.84–0.90) from 91 to 120 days. Similar protective effects were noted regardless of cardiovascular risk level in people over age 65 years. However, a greater protective effect was noted in those ages 40–64 years with low cardiovascular risk.

The frequency of receiving influenza vaccination may be important as several studies have noted a dose effect on the strength of protection [42, 50]. In a case control study of patients over 65 years of age, ever‐vaccinated individuals in the current vaccination season had a reduced risk of ischemic stroke admissions with an OR = 0.76 (95% CI 0.60–0.97). There was a significant trend of decreasing risk of ischemic stroke with an increasing number of vaccinations with an OR of 0.56 (95% CI 0.38–0.83) for the group with five vaccinations. Finally, several studies demonstrated vaccine effect outside of influenza season suggesting that preventing the infection itself can only be part of the explanation for the benefit of vaccine [38]. While this observation may be consistent with “healthy user” bias in the vaccine group, it is possible that there may be multiple mechanisms by which influenza vaccine exerts beneficial effects [41]. Possible mechanisms of protection include vaccine‐induced activation of the immune system, anti‐inflammatory effects by reducing TNFα, IL‐10 and increasing exhaled nitric oxide, prevention of secondary bacterial infections that may weaken the immune system, and stabilizing atherosclerotic plaque [47].

Despite improved methodologies, observational studies remain susceptible to bias. The only definitive way to answer the question about the effect of influenza vaccination and risk of stroke is by a randomized clinical trial (RCT), but presently, RCTs have only evaluated composite outcomes for major adverse CVEs, not specifically stroke. A meta‐analysis of five RCTs involving 9059 patients assessed for major CVEs estimated a pooled RR of 0.64 (95% CI 0.48–0.86). Because the data are derived from RCTs, this meta‐analysis provides compelling evidence of adjunctive role of influenza vaccination to prevent cardiovascular disease [47].

In addition, several systematic reviews and meta‐analyses combining observational and RCT data have been conducted to further clarify the role of influenza vaccination and risk of stroke [37, 44, 45, 49]. The impact of influenza, herpes zoster, and pneumococcal vaccine on CVE in older adults was evaluated in a systemic review published in 2023 [49]. Overall, the effect on CVE risk was beneficial in 28 of 33 studies evaluating influenza vaccine. Moreover, dual influenza and pneumococcal vaccination further lowered the risk of CVE, which includes stroke, congestive heart failure, ischemic heart disease, and myocardial infarction. A meta‐analysis of studies specifically evaluating the risk of stroke associated with influenza vaccination was performed by Tavabe et al. with a review period from 1980 to 2021 [44]. Overall, 14 studies were included in the analysis and demonstrated that influenza vaccination was associated with 16% reduction in risk of hospitalization for stroke (Table 1).

**TABLE 1:** Characteristics of meta‐analysis of studies evaluating influenza vaccination and risk of hospitalization for stroke.

Author	Year	Location	Study design	Sample size	OR	95% CI	% weight
Ohmit and Monto [51]	1995	US	Case–control	4487	1.17	0.73–1.88	2.26
Lavellee et al. [52]	2002	France	Case–control	689	0.37	0.15–0.87	0.73
Nichol et al. [36]	2003	US	Cohort	146,328	0.77	0.66–0.89	9.86
Smeeth et al. [7]	2004	UK	Self‐case control series	23,188	0.86	0.76–0.97	11.26
Wang et al. [53]	2004	Taiwan	Case–control	21,347	0.96	0.79–1.17	7.76
Grau et al. [54]	2005	Germany	Case–control	740	0.46	0.28–0.77	2.02
Puig‐Barbera et al. [55]	2007	Spain	Case–control	380	0.07	0.01–0.48	0.16
Pinol‐Ripoll et al. [56]	2008	Spain	Case–control	786	1.02	0.77–1.36	4.99
Hung et al. [57]	2010	Hong Kong	Cohort	36,636	0.67	0.54–0.83	7.06
Lin et al. [50]	2014	Taiwan	Case–control	3120	0.80	0.64–0.98	7.12
Siriwardena et al. [58]	2014	UK	Case–control	72,202	0.80	0.76–0.84	14.73
Lavallee et al. [43]	2014	International	Cohort	22,837	1.01	0.88–1.17	10.21
Vamos et al. [59]	2016	UK	Cohort	124,503	0.82	0.67–1.00	7.61
Chang et al. [60]	2020	Taiwan	Cohort	2,741,403	0.88	0.83–0.94	14.24
Overall					0.84	0.78–0.90	

*Source:* Adapted from Tavabe et al. [44].

Although some uncertainties remain about the effect size, number of vaccinations needed, and subgroups deriving the most benefit, the bulk of evidence indicates that influenza vaccination is associated with a significantly lower risk of stroke. Consistent with these observations, the American Heart Association recommends influenza as a secondary stroke prevention strategy in patients with vascular disease [61]. Since many middle‐aged and older adults have risk factors for cardiovascular disease, the benefits of influenza vaccine should be stressed by health care providers for all adults.

## 6 Microbes and Dementia

Dementia represents a group of diseases that affect memory, reasoning, personality, mood, and behavior. The most common cause of dementia is ad although there are multiple other causes of dementia such as vascular, Lewy body, frontotemporal, and Parkinson's‐related dementia [62]. It is estimated that nearly 7 million adults are currently living with dementia and by 2060, the number is projected to be nearly 14 million [63]. ad was first described by Alois Alzheimer in 1907, and the neuropathology is marked by (amyloid beta) Aβ proteins in plaques outside neurons and neurofibrillary tangles composed of hyperphosphorylated Tau protein inside neurons. These changes result in loss of synapses and neurodegeneration [64]. There are multiple hypotheses as to the cause of ad, and genetics and environment are felt to play a role [65]. The concept that ad might be linked to infection was first proposed in 1907 by Oskar Fischer, but more sustained first efforts to identify a microbial connection and neurodegenerative diseases began in the 1980s. The founding of the microbial hypothesis of ad began in 2016 when Itzhaki published an editorial outlining the evidence and case for a causal role of pathogens in [66]. There is a growing body of evidence that viral infection is linked to ad, particularly herpes viruses and HIV and other viral infections including influenza [3].

**FIGURE 1:**
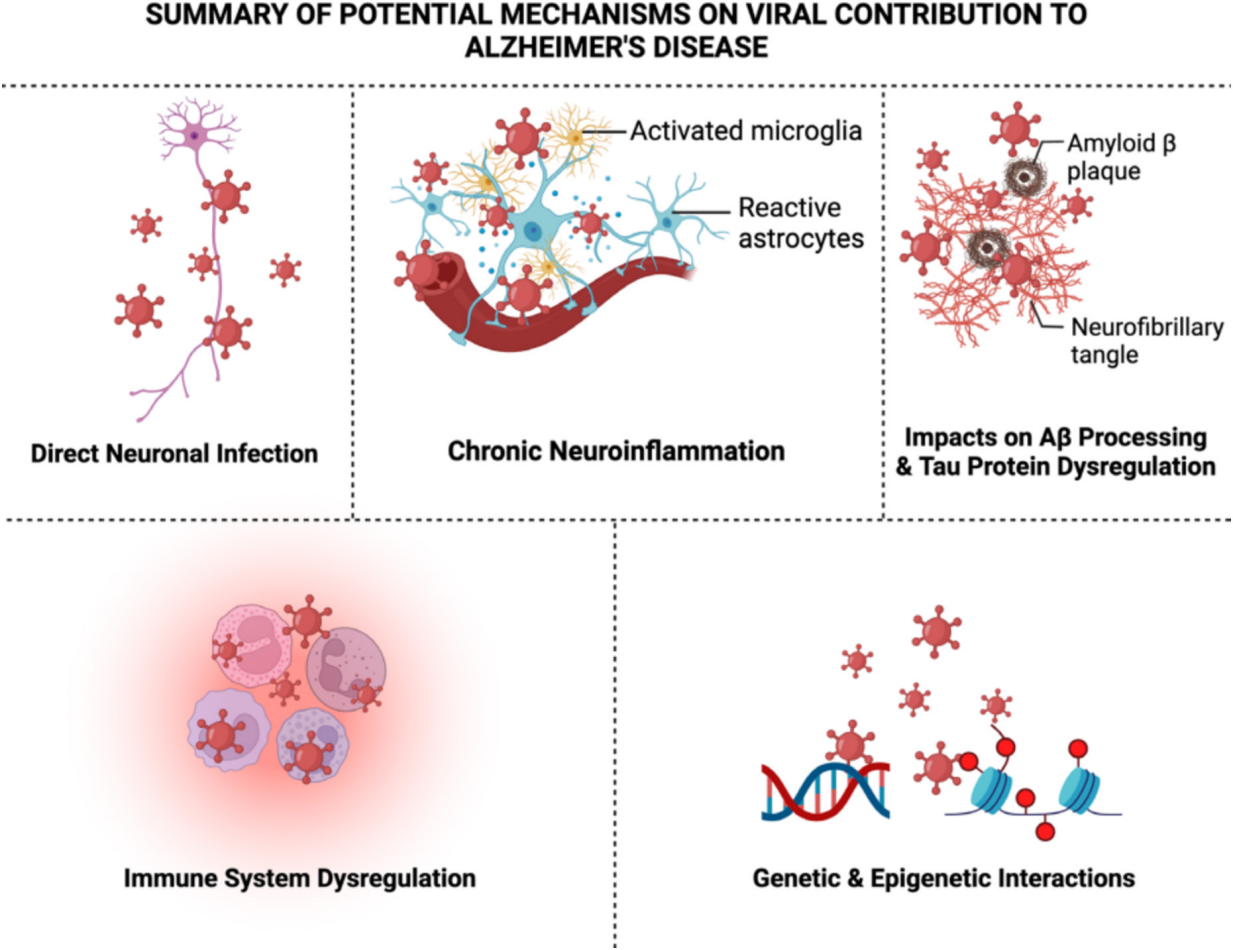
A summary of the potential mechanisms of viral effects associated with Alzheimer's disease (ad). *Source:* From Rippee‐Brooks et al. [65].

Evidence for infectious hypotheses includes postmortem studies, epidemiologic studies, genetic data, and preclinical studies (Figure 1). It postulated that viral effect may be direct or indirect [64]. With direct effect, the virus enters the brain and causes neuronal death or activates antiviral responses leading to neuroinflammation and ad pathology. With the indirect hypothesis, it is presumed that pathology is mediated by systemic inflammation.

Herpes viruses and HIV are well known to enter the central nervous system and cause direct viral damage. Most influenza viruses are not neurotropic, and thus, mechanisms in neurodegenerative disease are more likely indirect. However, influenza H5N1 has been implicated in the phosphorylation and aggregation of α‐synuclein, which plays a role in Parkinson's disease and Lewy body dementia [67]. In addition, there is structural similarity between residue 42 of Aβ and influenza hemagglutinin (HA), suggesting a mechanism by which influenza might exert neurotoxicity [68, 69]. Seasonal H1N1 does not cross the blood brain barrier, but nonetheless, can lead to microglial overactivation [67]. Jang et al. demonstrated increased levels of proinflammatory cytokines in the brains of mice inoculated intranasally with H1N1 virus with some mirroring the inflammatory cytokines found in the lungs and but other cytokines unique to the brain [23].

Although provocative animal data exists, epidemiologic studies linking influenza infection to subsequent development of ad are very limited. In a large observational case–control study in the United Kingdom, Imfeld et al. matched 19,463 patients who developed ad between 1998 and 2013 with patients without dementia based on GP encounter coding. No association of risk of ad was found with any prior influenza illness, number of influenza infections, severity of influenza, or influenza in the presence of comorbid conditions. However, a more recent study using data from a Finnish biobank and validation with an independent database in the United Kingdom, investigators found associations with a variety of virus exposures and neurodegenerative diseases [9]. Of 45 viral exposures identified as significantly associated with neurologic disorders in the Finnish database, 22 were replicated in the UK database. Influenza with pneumonia was significantly associated with dementia, vascular dementia, Alzheimer's dementia, Parkinson's disease, and ALS. When lag times were introduced, the highest hazard ratios were within 1 year, followed by 1 to 5, then 5–15 but remained significant with all time frames.

If cognitive impairment is associated with influenza infection, it may be related to severe illness with the complications therein. Although not influenza specific, Girard and colleagues examined long‐term cognitive impairment after hospitalization for community‐acquired pneumonia (CAP) [70]. Cognitive function was shown to be significantly impaired up to 12 months after hospitalization for CAP. Deficits were most common in visuospatial function, attention, and memory. Three other studies of cognitive function and pneumonia found similar results with significantly increased rates of dementia following hospitalization [71, 72, 73]. Of note, interpretation of the above‐mentioned studies should be cautious since cognitive declines observed may be due to preexisting dementia, which has been revealed rather than being causally linked with an acute respiratory illness.

Since influenza infection is universal throughout life and dementia is fortunately not, if there is a relationship between influenza and dementia, it is likely complex and multifactorial.

## 7 Impact of Vaccination on the Development of AD

**FIGURE 2:**
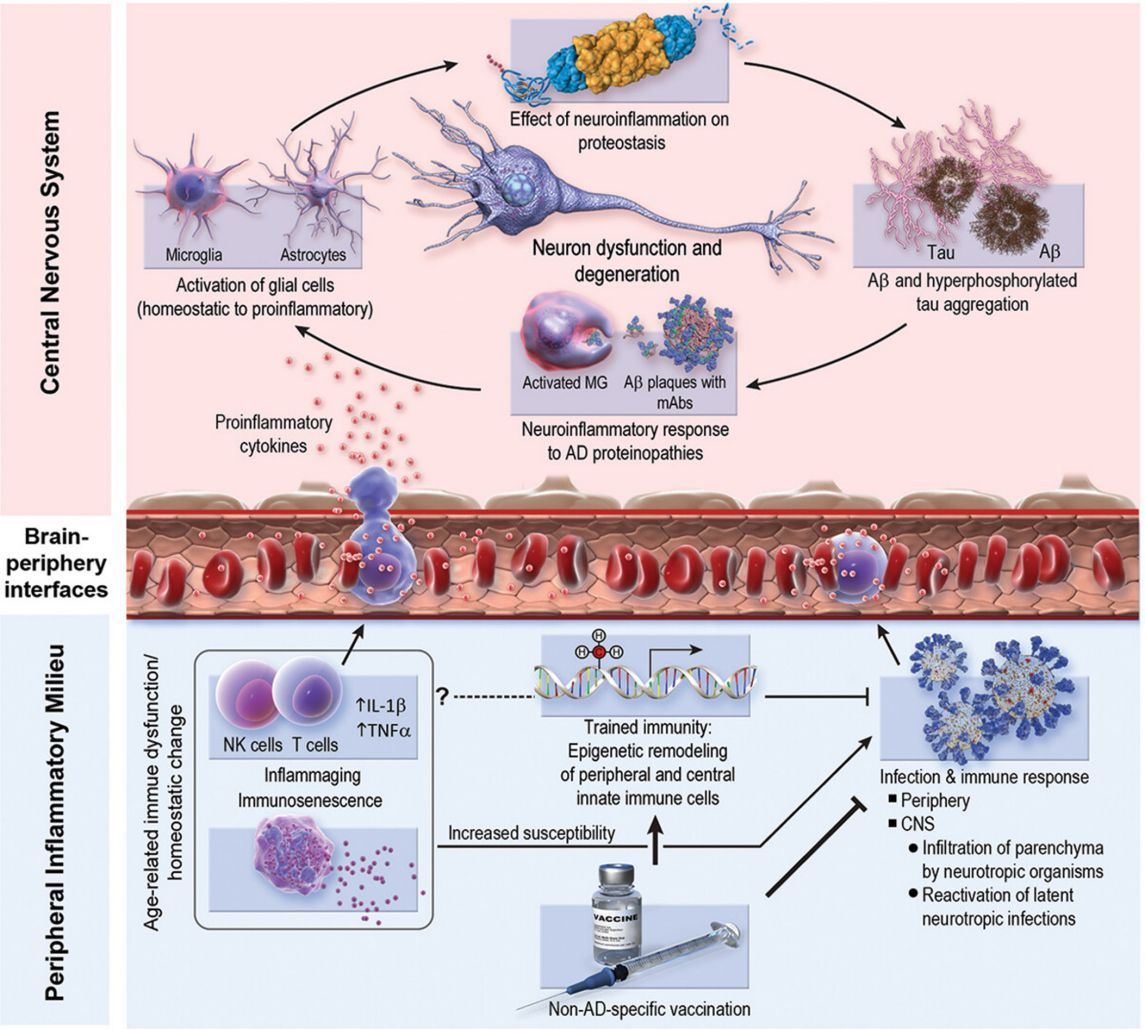
Possible mechanisms to explain the protective effects of vaccination on risk of dementia. *Source:* From Bukhbinder et al. [74].

ad is a pathologically complex disease with multiple contributing factors, and thus, treatment and prevention will likely require multifaceted approaches. There is now accumulating evidence that vaccines exert a protective effect on the risk of developing AD [74]. The potential mechanisms of protection include reduced risk of infection with its resultant harmful effects, improved immune‐mediated clearance of Aβ plaques, and modulation of the normal inflammatory response to the ad pathology that results in damage to normal surrounding brain (Figure 2).

In the first study evaluating vaccination and dementia risk, investigators analyzed past exposures to routine vaccines and risk of developing ad in people participating in a prospective Canadian Study of Health and Aging [75]. Adults over age 65 years were enrolled between 1991 and 1992 with baseline screening and clinical evaluations and completed follow‐up 5 years later with repeat evaluations. Of 3682 participants, 183 subsequently developed ad. After adjustment for clinical confounders, past exposure to vaccines significantly decreased risk of ad (diphtheria, tetanus, and polio) whereas a nonsignificant trend towards protection was noted with influenza vaccine.

Over a decade passed before interest was revived regarding the effect of vaccination on dementia and two studies evaluated influenza vaccine in high‐risk populations in Taiwan. Liu et al. used data from the Taiwan National Insurance Database and included 5745 chronic kidney disease (CKD) patients who were vaccinated and 6198 patients who were never vaccinated for influenza. Patients were followed for 6 years, and logistic regression was used to estimate effect of vaccination while accounting for multiple clinical cofounders. Influenza vaccination was significantly associated with a lower risk of dementia during influenza season, nonseason, and all seasons with HRs of 0.68, 0.58, and 0.64, respectively. The association remained significant for all clinical subgroups regardless of age or comorbidities and exhibited a dose‐dependent effect on the reduction of dementia. Luo et al. evaluated the Taiwanese database for risk of dementia related to influenza vaccination in chronic obstructive pulmonary disease (COPD) patients over age 60 years between 2001 and 2012 [76]. Multivariate analysis was used to calculate HR of dementia in relation to influenza vaccination, and the number of vaccines was categorized as none, 1, 2–3, and > 4. The adjusted HR of dementia was 0.68 (95% CI 0.62–0.74) when comparing vaccinated to unvaccinated persons. There was a trend towards a dose effect with those having a history of > 4 vaccination demonstrating a HR of 0.44 (95% CI 0.40–0.50).

Investigators in Houston, Texas, have carried out several analyses of influenza vaccination and risk of ad. Amran et al. used electronic health records to analyze approximately 300,000 propensity score matched vaccinated and unvaccinated persons over age 60 years [77]. Survival analysis was performed on the entire cohort with development of ad as the outcome. Receipt of influenza vaccination was associated with decreased prevalence of ad with an OR of 0.83, *p* < 0.0001 with the frequency of vaccination also having a significant effect. This group extended their observations to 935,887 influenza vaccinated and unvaccinated matched pairs followed from 2009 to 2019. During follow‐up, 5.1% of vaccinated and 8.5% of unvaccinated persons developed ad resulting in a RR of 0.60 (95% CI 0.59–0.61) [68]. Finally, these investigators have begun a trial of high dose or adjuvanted influenza vaccine compared to standard influenza vaccine, postulating that the protective effect against ad would be stronger with high dose or adjuvanted vaccines [74]. This study design requiring all subjects be vaccinated should mitigate somewhat against healthy user bias since all subjects are vaccinated. Preliminary data indicate greater protection against ad in the high dose group compared to standard vaccine.

Dementia risk following influenza vaccination was also assessed in large veterans' cohort from 2009 to 2019 for patients 65 years and older and free of dementia for 2 years [78]. Propensity scores and inverse probability of treatment weighting were used to control for confounders. After controlling for confounders, vaccinated persons had reduced risk of dementia with a HR of 0.86 (95% CI 0.83–0.88). Notably, the effect was only significant for those receiving six or more influenza vaccinations.

The results of multiple studies of influenza vaccine and dementia risk were addressed in a systematic review and meta‐analysis conducted by Veronese et al. [79]. The review period was up to September 2021, and the meta‐analysis included five studies for a total of 292,157 older adults free of baseline dementia and followed for a mean of 9 years. Overall, influenza vaccine decreased the risk of dementia with a RR of 0.97 (95% CI 0.94–1.00). After adjusting for confounders, the RR was 0.71 (95 CI 0.60–0.94) resulting in approximately a 29% reduction in dementia risk.

Several investigators have looked at the effect of other routine vaccinations on the risk of dementia including tetanus, Tdap, Zoster, or pneumococcal vaccine as well as influenza and demonstrated risk reductions for ad and dementia in general [80, 81]. A meta‐analysis of any adult vaccine and risk of ad was conducted by Wu et al. in which 17 studies were selected with nearly 2 million people in the analysis [82]. Overall, pooled results showed that vaccinations were associated with a 35% lower risk of dementia with a HR of 0.65 (95% CI 0.60–0.71). All vaccines showed a trend towards benefit with significant reduction for rabies, Tdap, Zoster, Hepatitis A and Hepatitis B, Typhoid, and influenza.

Notably, people with more vaccine types and more annual influenza vaccines were less likely to develop dementia.

Finally, a study of common adult vaccines and risk of dementia by Douros et al. took a different approach starting by matching dementia patients with nondementia patients and looking back at vaccine use [83]. Investigators in the United Kingdom assembled a dementia free cohort and used a nested case–control study, matching each patient with dementia to four nondementia patients. Using a conditional logistic regression, adjusted OR of dementia was calculated compared to nonvaccine exposure using 2‐year lag period such that dementia onset in the 2 years after vaccine was not counted. Contrary to other studies, exposure to vaccines was associated with increased risk of dementia with an OR of 1.38 (95% CI 1.36 to 1.40). This risk appeared to be driven by influenza, OR 1.39 (95% CI 1.37 to 1.41), and pneumococcal vaccine, OR 1.12 (95% CI 1.11 to 1.113). The authors note that while an increase in dementia caused by vaccination seemed unlikely, their study points out how methodology and various types of bias can significantly affect study results.

Given the problems interpreting the results of clinical studies, data from animal studies may yield important clues. The APP/PS1 mouse model recapitulates ad pathology and can be used to study interventions to treat or prevent ad. Yang et al. assessed spatial learning and memory, brain plaque burden, activated microglia cells, cytokines in blood and brain, and proportion of Tregs in the spleen in influenza‐vaccinated mice [84]. Mice were given five monthly injections of influenza vaccine, which appeared to activate microglia, reduce Aβ burden, and improve clinical performance of mice. Restoration Tregs seemed to abrogate the protective effects of vaccine.

Several features are common to the observational studies and include a protective effect during noninfluenza seasons, a dose effect (more is better), and protective effects across multiple vaccine types. While some may suggest that these findings indicated unmeasured bias, it also possible that nonspecific immunomodulation is an important factor. Clearly, more research with careful attention to study design and potential sources of bias are needed in this field.

## 8 Conclusion

Influenza results in approximately 1 billion infections each year leading to 3–5 million hospitalizations and 290,000–650,000 deaths worldwide. Stroke and ad and other dementias are also leading causes of death globally. The intersection of these two important diseases is now well established (Table 2). Given that influenza is a vaccine‐preventable disease, every effort should be made to encourage annual vaccination. While stroke is now recognized as a complication of influenza, the association of influenza infection with ad or other forms of dementia is less clear. The possibility that influenza vaccine might reduce the risk of dementia is intriguing, and multiple studies are now suggesting benefit although conclusive evidence remains elusive. However, given the devasting effects of dementia, more research is clearly needed regarding the possible protective effects of influenza and other vaccines to reduce risk of dementia.

**TABLE 2:** Summary of major neurologic syndromes complicating influenza infection.

Neurologic events	Comments	Vaccine effect
Acute complications		
Delirium	Common in elderly	Not specifically studied^a^
Seizures	Most common in infants	Not specifically studied^a^
Meningitis‐encephalitis	Rare	Not specifically studied^a^
ADEM	Rare	Not specifically studied^a^
GBS	Rare	Not specifically studied^a^
Chronic complications		
Parkinson's disease	1.7–2‐fold increase in OR in 10 years following influenza	One study—no effect
Stroke	3–10‐fold increase in OR first 7 days with increased risk out to 28 days	Multiple studies showing benefit. Reduction in OR of 0.56 to 0.76 for stroke
Dementia	Increased rate of any dementia, vascular dementia and Alzheimer's disease 1–15 years after influenza with strongest effect 1 year after infection	Conflicting data. Meta‐analysis indicated overall 35% reduction in dementia risk with multiple vaccines. Single study showing increased OR (1.4) of dementia with influenza vaccine.

Abbreviations: ADEM, acute disseminated encephalomyelitis; GBS, Guillain Barre Syndrome; OR, odds ratio.

^a^ Not specifically studied but overall vaccine efficacy (VE) to prevent influenza infection would be expected to offer protection. VE varies with the age and health of the host and match of the vaccine and circulating strain.

## Author Contributions

**Ann R. Falsey:** conceptualization, writing – review and editing, writing – original draft.

## Conflicts of Interest

The author declares no conflicts of interest.

### Peer Review

The peer review history for this article is available at https://www.webofscience.com/api/gateway/wos/peer‐review/10.1111/irv.70071.

## Data Availability

This is a review article and there are no data to make available.
